# Novel Method for Restitution of a Torqued and Entrapped Kinked 7F Catheter in the Ascending Aorta

**DOI:** 10.7759/cureus.49856

**Published:** 2023-12-03

**Authors:** Ankit Gupta, Bhushan Shah, Ashish Jain

**Affiliations:** 1 Department of Cardiology, All India Institute of Medical Sciences, Raebareli, IND; 2 Department of Cardiology, All India Institute of Medical Sciences, Bhopal, IND

**Keywords:** aorta, transfemoral, knot, kink, catheter

## Abstract

Knotting and kinking of diagnostic coronary artery catheters are potentially catastrophic complications though their occurrence is uncommon. However, abrupt kinking of 7F guide catheters in the ascending aorta becomes a quirky puzzle. This case emphasizes the importance of avoiding kinking and provides recommendations for catheter retrieval in the unlikely event of this complication. To the best of our knowledge, the technique used in our case has not been described before.

## Introduction

With the passing years, the number of patients requiring cardiac catheterization has been escalating. With this increasing number of diagnostic as well as therapeutic cardiac interventions, the incidences of varied peri-procedural complications have also risen. Some of such complications are catheter fracture, knotting, or kinking. Catheter fractures or kinks are infrequent occurrences in catheterization laboratories, but when they do happen, they pose terrible challenges that can hinder the successful completion of the procedure [1].

Catheter kinking is observed to occur more frequently when engaging the right coronary ostium, particularly in cases of a tortuous aorta [2]. This phenomenon has been attributed to increased torque build-up in the catheter's proximal section during manipulations, as opposed to the distal end, when navigating through vessels with significant tortuosity [3]. Here, we present a case of the entrapped kinked 7F catheter in the ascending aorta. This case also highlights the significance of preventing kinking and offers recommendations on how to retrieve the catheter in the rare event of such complication.

## Case presentation

**Figure 1:**
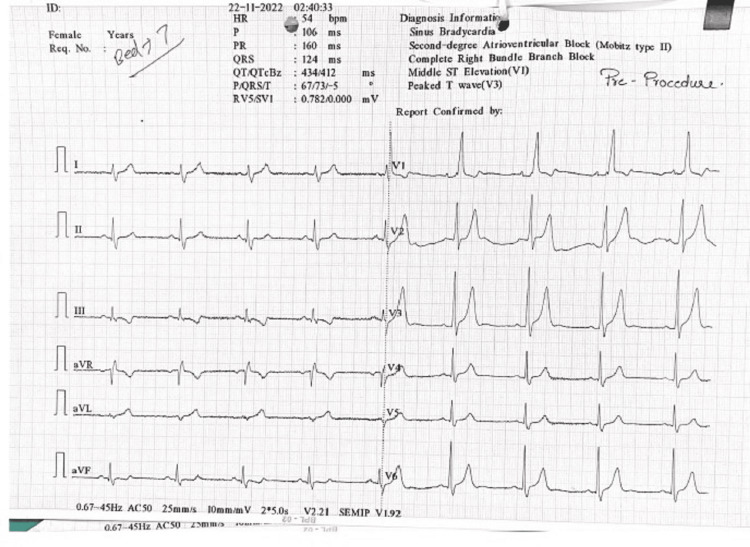
Electrocardiogram at initial presentation

A 50-year-old male presented with angina-like symptoms for five days. Physical examination was pertinent for blood pressure of 138/70 mm Hg, heart rate of 68 beats/min, oxygen saturation of 97% on room air, regular cardiac rhythm, and intact distal pulses. He was sent for an echocardiogram that demonstrated a left ventricular ejection fraction of 56-60%, at rest. ST elevation and peaked T wave were observed in Inferior leads (Figure 1).

Troponin levels were raised. In view of raised troponin levels, he was taken up for diagnostic coronary angiogram via the right radial route which revealed significant distal right coronary artery-posterior descending artery/posterolateral vein (RCA-PDA/PLV) bifurcation stenosis (90% stenosis [medina class:1,1,1]) (Video 1). It was planned to perform bifurcation with the two-stents technique at RCA-PDA/PLV, for which the patient provided written and informed consent.

**Video 1:** Right coronary artery-posterior descending artery/posterolateral vein (RCA-PDA/PLV)

Stenting to RCA bifurcation was planned via the femoral approach. A 7-F Judkins right 4 (JR4) catheter was advanced over a 0.035-inch guide wire into the ascending aorta and engaged with right sinus. While manipulating the catheter suddenly the guide catheter got kinked abruptly and severe torque was noted in the ascending aorta like padlocked (Video 2).

**Video 2:** Kinked 7F guide catheter entrapped in the ascending aorta

However, multiple attempts failed to untwist the kinked coronary catheter by counter-rotating the catheter. The kinked guide catheter was stuck in the ascending aorta in the current case so pulling or giving mechanical stress to the catheter may break the catheter or can block the femoral artery in that multiple torque & folds. Unfortunately, the gooseneck snare was unavailable that day. Therefore, a handmade single snare was prepared immediately by using a 5F Judkins right (JR) diagnostic catheter, making a single-loop snare with the tip of coronary wire and a small inflation of 2 X 8 mm balloon at the tip of 5F catheter (Video 3).

**Video 3:** Handmade single-loop snare

The handmade single loop snare was inserted through the right radial route; after multiple efforts the tip of the kinked coronary catheter in ascending aorta was snared (Video 4), tightly pulled towards the right subclavian artery, and straightened, and the kinked catheter was unfolded (Video 5), which was followed by untwisting the torqued part (Video 6).

**Video 4:** Snaring the tip of the kinked entrapped catheter

**Video 5:** Straightening and unfolding the catheter via the right radial route

**Video 6:** Untwisting of the straightened catheter

**Figure 2:**
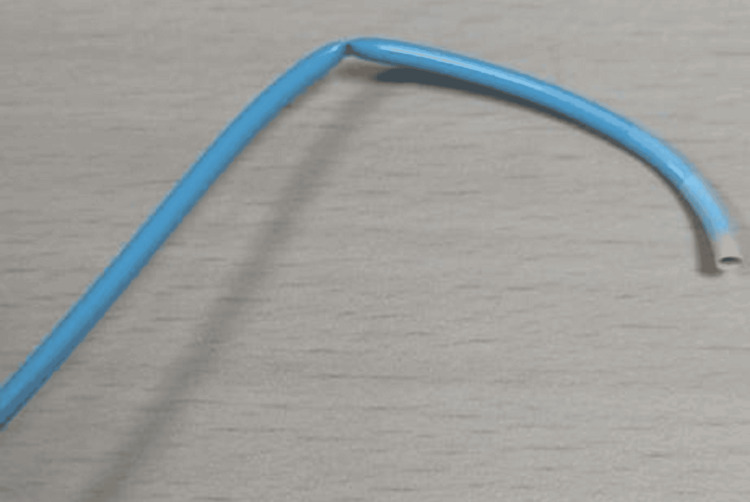
Kinked 7F guide catheter entrapped in the ascending aorta and retrieved

Finally, we were able to place the PTFE Bard Guide Wire 0.035 x 150cm J Tip wire across the kinked and torque part of the guide 7F catheter (Video 7), and the catheter was retrieved via the femoral route (Figure 2, Video 8).

**Video 7:** Straightened untwisted 7F catheter secured by placing J Tip Guide Wire 0.035

**Video 8:** Retrieved 7F catheter

Subsequently, RCA was engaged with the 7F JR 3.5 guiding catheter, and after pre-dilatation, a 2.5 x 16 mm Tetriflex sirolimus-eluting stent (Sahajanand Medical Technologies Limited, India) was deployed in PLV and a 2.75 x 20 mm Tetriflex SES was deployed in PDA using the mini culotte technique. After kissing balloon dilatation and performing the proximal optimization technique [4], the thrombolysis in myocardial infarction (TIMI) III flow was achieved without any further complications.

## Discussion

Diagnostic catheter knotting and kinking are uncommon but potentially serious vascular complications. Overall, it is 90% common in the transradial route because its tortuosity is like that of brachial as well as subclavian artery. The transfemoral approach is currently popular and safest for vascular access during percutaneous coronary intervention (PCI). Most interventional cardiologists are more familiar with doing the complex PCI procedure of the patient via the transfemoral route. Similarly, we planned to do RCA bifurcation PCI with the two-stent technique via right femoral access, since the transfemoral approach is more convenient for manipulating the devices, including the guide catheter during complex anatomy in diagnostic as well as therapeutic cardiac interventions. Yet, the catheter got kinked in the ascending aorta. The retrieval of such a catheter via the femoral artery route should be executed with great caution and precision, as any excessive force applied could potentially result in damage to the major arteries, leading to severe complications [5].

It is well said that “prevention is better than cure”; such that during catheterization, it is better to prevent catheter kinking/knotting by keeping in mind various strategies like avoiding aggressive catheter torquing, considering long sheath, maintaining a guidewire through the catheter, considering left radial approach in elderly patients [6].

The available literature also discusses a range of techniques for retrieving coronary catheters that have become kinked or trapped. As initial steps, straightforward maneuvers such as gentle pulling, rotation in opposite directions, and advancing a guidewire can be employed to address minor kinking [1,7]. However, these basic techniques may not suffice for complex loops and severe kinks. In cases where these initial methods prove ineffective, a series of maneuvers can be considered: Inflating a blood pressure cuff on the same-side brachial region, followed by gently untwisting the catheter; using a Goose Neck Snare to capture the catheter's tip, followed by untangling any knots within a large-caliber vessel; employing an EN Snare catheter to grasp the distal tip of the kinked catheter and then twisting the distal and proximal ends in opposite directions to unravel any knots; cutting the catheter's hub, placing a long sheath over it, and pulling the kinked segment into the sheath to straighten it; employing balloon-assisted trapping and removing the kinked catheter through a large-bore sheath [6]. These techniques provide a range of options for safely and effectively addressing kinked or entrapped coronary catheters, based on the specific clinical scenario.

In the present case, the catheter was initially inserted through the femoral artery and got kinked. Subsequently, for its restitution, a handmade snare was inserted through the radial route. On contrary in terms of access routes, Khoubyari et al. reported a case where a catheter became entrapped and kinked in the radial artery, but it was effectively extracted using a gooseneck snare through the transfemoral approach. Subsequently, both the distal and proximal ends of the catheter were rotated in opposite directions simultaneously, facilitating the straightening and retrieval of the catheter [8]. Likewise, Rafie et al. documented an incident where a JR4 catheter got knotted within the left common iliac artery. Following unsuccessful attempts to remove the coronary catheter using standard procedures, they resorted to utilizing the Amplatz Goose Neck snare catheter, accessed through the right femoral approach, to successfully retrieve the knotted catheter [5].

Various cases have been reported in the literature that utilized varied techniques for removal of knotted or kinked catheters. For instance, Patel et al. in their case applied a simple, non-invasive method to unkink the catheter by using a long ultrasound-guided supraclavicular insertion (USCI) sheath, which facilitated the smooth removal of the tangled catheter, enabling them to complete the procedure without any additional complications [2]. Malik et al. reported a first-of-its-kind case that applied balloon-assisted trapping for retrieval of a kinked catheter [6]. Subsequently, Takahagi et al. also prioritized an untangling technique using a balloon catheter and successfully untangled the knotted catheter. Moreover, Takahagi et al. also stated that the applicability of using a snare catheter was difficult because it requires a sufficiently large bore of the sheath to pass the knotted catheter [9].

The literature recommends that when handling a catheter during coronary angiography, the torque should be effectively transmitted to the catheter's tip. If this transmission is not achieved, it indicates that the catheter is twisted along the shaft, and the operator should cease manipulation to prevent catheter kinking [5]. If you encounter that the catheter has kinked, we recommend not to pull it back or twist it. The kinked catheter must be untwisted with the insert wire then the catheter tip should be snared and subsequently cut catheter encase with larger sheath. Cardiologists conducting coronary angiography should acquaint themselves with at least one variety of snare catheters, which should be readily accessible for retrieving wire or catheter fragments within the vascular system.

## Conclusions

Preventing complications is typically a simpler task than managing with them once they arise. It is crucial to exercise caution in all stages of catheter manipulation, particularly within complex and tortuous blood vessels. A rapid assessment of the potential advantages and disadvantages should be conducted when considering strategies to address knotted or entrapped catheters. Regular maneuvers to unknot the catheter might be unsuccessful. We used a self-made single loop snaring technique by radial access to fix the distal tip of the catheter and then reshaped the catheter by smooth rotation.
